# Scents modulate anxiety levels, but electroencephalographic and electrocardiographic assessments could diverge from subjective reports: a pilot study

**DOI:** 10.3389/fnbeh.2025.1534716

**Published:** 2025-09-16

**Authors:** Marina Morozova, Irina Gabrielyan, Daria Kleeva, Victoria Efimova, Mikhail Lebedev

**Affiliations:** ^1^Center for Neurocognitive Research (MEG Center), Moscow State University of Psychology and Education, Moscow, Russia; ^2^Vladimir Zelman Center for Neurobiology and Brain Rehabilitation, Skolkovo Institute of Science and Technology, Moscow, Russia; ^3^Peoples Friendship University of Russia (RUDN University), Moscow, Russia; ^4^MSU Institute for Artificial Intelligence, Lomonosov Moscow State University, Moscow, Russia; ^5^Research Center in the Field of Artificial Intelligence, Lomonosov Moscow State University, Moscow, Russia; ^6^Herzen State Pedagogical University of Russia, Saint Petersburg, Russia; ^7^“Prognoz” Children’s Neurological Clinic, Saint Petersburg, Russia; ^8^Faculty of Mechanics and Mathematics, Lomonosov Moscow State University, Moscow, Russia; ^9^Sechenov Institute of Evolutionary Physiology and Biochemistry of the Russian Academy of Sciences, Saint Petersburg, Russia

**Keywords:** anxiety, dentistry, aromatherapy, hyraceum, lavender, EEG, HRV, STAI

## Abstract

Scents can influence anxiety, including that experienced in clinical environments. This study examined the effects of two distinct aromas: lavender, a fragrance widely recognized for its calming properties, and African stone, a musky and relatively unfamiliar scent. Twenty healthy participants underwent alternating periods of rest and scent inhalation in a dental office environment while anxiety was assessed using the State–Trait Anxiety Inventory (STAI), electroencephalographic (EEG) measures of theta, alpha, and beta power ratios, and electrocardiographic (ECG) measures of heart rate variability (HRV). Lavender inhalation significantly reduced self-reported state anxiety scores but did not produce measurable changes in EEG or HRV indices, possibly due to the short (5 min) exposure duration. African stone, in contrast, did not alter self-reported anxiety but induced significant physiological effects, including reduced theta and, increased alpha power in parieto-occipital regions, and decreased high-frequency (HF) and total HRV power. While the EEG changes are consistent with a more relaxed state, the HRV reductions could indicate a heightened autonomic arousal, suggesting that African stone could have triggered increased attention and physiological activation rather than merely relaxation. These findings demonstrate a divergence between subjective and physiological responses to scent exposure. Lavender appears to primarily reduce perceived anxiety, while African stone influences physiological arousal. We suggest that a multimodal approach be applied in aromatherapy research.

## 1 Introduction

Anxiety is a common challenge for patients visiting medical offices. In dental settings, the sight of clinical tools and the anticipation of discomfort often cause anxiety to rise sharply. Aromatherapy offers a promising way to ease this tension by influencing emotional states and calming the body’s stress response. Anxiety typically triggers increased activity of the sympathetic nervous system, leading to measurable changes in physiological arousal. These changes can be captured through electroencephalography (EEG) and electrocardiography (ECG) recordings. This study explored how two different scents affected anxiety levels in healthy participants placed in a dental office environment. Alongside physiological data from EEG and ECG, participants’ self-reported anxiety scores provided information on their subjective experience of the scents’ calming effects.

EEG recordings offer objective insights into brain activity patterns associated with different emotional states, including anxiety and relaxation. EEG responses to anxious conditions have been linked to increased beta and theta power in the frontal cortex (Cavanagh and Shackman, 2014; Giannakakis et al., 2015), reduced asymmetry index (Muhammad and Al-Ahmadi, 2022), and reduced alpha power (Vanhollebeke et al., 2022). Increased beta and reduced alpha power can be signs of heightened alertness associated with anticipatory stress (Vanhollebeke et al., 2022). By examining changes in EEG patterns during exposure to scents, it is possible to assess how specific odors affect neurological mechanisms of anxiety. For example, reductions in beta and increases in alpha activity in response to an odor would indicate reduction in anxiety and better relaxation. Previous studies have employed EEG rhythms and evoked potentials to assess olfactory-induced responses (Sowndhararajan and Kim, 2016), including representation of such characteristics as odor pleasantness (Becerra et al., 2018) and attention to scents (Morozova et al., 2024). Furthermore, we have used odors as neurofeedback of cortical activity (Medvedeva et al., 2024; Ninenko et al., 2024). Certain scents, such as lavender, have been reported to enhance alpha and theta activity (Sayorwan et al., 2012).

As a supplement EEG assessment, ECG provides a measure of the autonomic response to anxious conditions. Anxiety and stress-related disorders are associated with increased heart rate and decreased heart rate variability (HRV) (Appelhans and Luecken, 2006; Chalmers et al., 2014; Kim et al., 2018; Yeragani et al., 1993). There is a general consensus that the high-frequency HRV (HF, 0.15–0.4 Hz) is associated with respiratory sinus arrhythmia and represents an index of parasympathetic modulations. Conversely, there is no agreement on the interpretation of the low-frequency (LF, 0.04–0.15 Hz) HRV. LF HRV power has been traditionally interpreted as an index of sympathetic modulations. However, a recent clarification indicates that LF HRV represents baroreflex-mediated modulations of both sympathetic and parasympathetic inputs to the heart (Goldstein et al., 2011; Huang et al., 2022; Shinba et al., 2024). Therefore, HRV indices should be interpreted cautiously, considering the multitude of autonomic control mechanisms they depend on. Notably, a previous study has shown that soothing scents, such as lavender, increase the HF of HRV and promote relaxation (Duan et al., 2006).

Calming scents could be incorporated into anxiety-inducing environments, such as dental offices, to alleviate patient anxiety. Scents like lavender, orange, and peppermint have been suggested for use in medical and driving settings to reduce stress and fatigue (Czakert et al., 2024; Jiang et al., 2024; Lehrner et al., 2000). The current study adds to these works an assessment of EEG and ECG responses to scents in a dental office environment. We investigated the differential impacts of three distinct stimuli: water as a control, lavender oil, and African stone (also referred to as hyraceum and noted for its musky scent).

Lavender oil is prominent in the literature as a commonly studied scent with calming effects (Donelli et al., 2019), so it was an obvious choice for this study. In contrast, the effects of African stone, an animal essence used in perfumery, are not well understood. African Stone, or hyraceum, is a fossilized excrement of the rock hyrax, and its scent can be described as animalistic with leather notes, similar to civet, musk, castoreum and ambergris. Hyraceum has been used in traditional South African medicine for various purposes, including treating epilepsy (Magama et al., 2018). Some studies have tested hyraceum samples for potential neuroactive properties, specifically affinity for GABA-benzodiazepine receptors (Olsen et al., 2007). Through comparative analysis of these two scents, we aimed to elucidate the nuanced effects of the olfactory stimuli on the level of anxiety and its neurophysiological and physiological correlates, contributing to a better understanding of the role of scents in emotional regulation.

## 2 Materials and methods

### 2.1 Subjects

Twenty healthy volunteers, 10 females and 10 males, aged 36.0 ± 9.3 years (mean ± standard deviation), all right-handed, participated in the study. Each participant attended one experimental session lasting approximately 90 min. All participants gave informed consent, and the study received approval from the Ethics Committee of the Skolkovo Institute of Science and Technology, Moscow. Exclusion criteria included any history of neurological disorders or significant alterations in olfactory function within the previous 6 months.

### 2.2 Experimental setup

**Figure 1:**
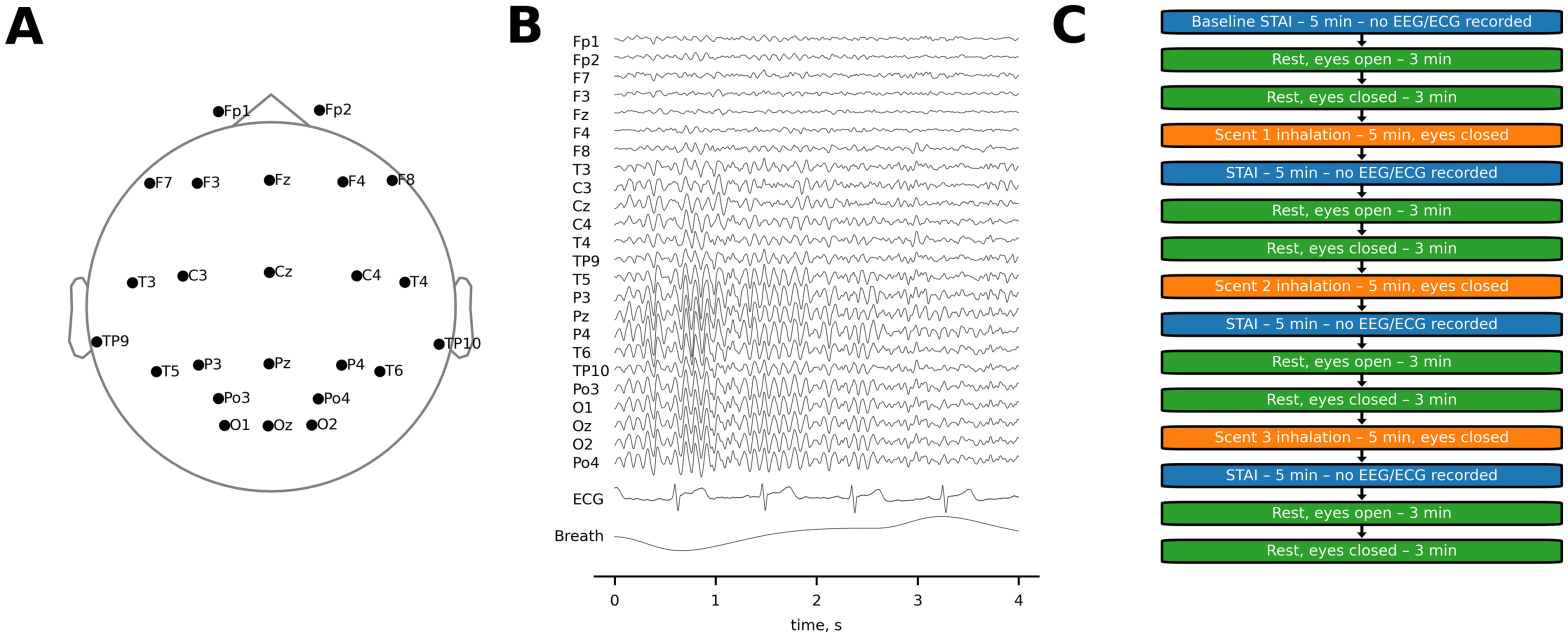
Experimental settings. **(A)** The layout of EEG recordings. **(B)** An example of the recorded EEG, ECG, and respiratory signals. **(C)** Schematics of the experimental protocol.

EEG data were collected using an NVX-36 amplifier (MKS, Russia). Recordings were made from 24 EEG channels following the international 10–20 system at a sampling rate of 500 Hz (Figure 1A). Ag/AgCl electrodes with electrode gel were used, with a monopolar montage referenced to the FCz electrode. Electrode impedance was kept below 15 kΩ to ensure signal quality (Figure 1B). ECG data were collected through a separate channel connected to the left collarbone, and respiration was measured using a nasal thermometric breath sensor (TRSens, MKS, Russia).

### 2.3 Study design

The influence of scent exposure on anxiety levels were tested in a dental office. All participants had a history of previous dental treatments and reported experiencing heightened anxiety during such procedures. They were informed prior to the experimental session that no dental interventions would be performed, so their possible anxiety was related to the realistic clinical environment rather than anticipation of treatment.

Three stimuli were tested: water (control), lavender oil, and African stone. Each stimulus was presented for 5 min in a randomized order, with participants inhaling the scent while their eyes were closed. Each participant was seated in a dental chair, and odorants were placed on a stand under the participant’s nose.

The participants completed the State–Trait Anxiety Inventory (STAI) prior to the experiment and immediately after each scent exposure, for a total of four STAI assessments. The STAI, a 40-item questionnaire, was used to measure both trait and state anxiety, with higher scores indicating greater anxiety. The STAI questionnaire took approximately 5 min to complete. After filling the STAI questionnaire, participants rested with their eyes open for 3 min, followed by 3 min resting with eyes closed. Thus, the intervals between scent presentations were approximately 11 min. Additionally, data were sampled during the 3-min resting periods prior to the very first and after the last scent presentation, allowing for baseline comparisons. The eyes-open condition, recorded immediately after taking the STAI questionnaire, was useful as a control for general alertness during the time intervals between the scents.

Thus, each session was composed of the initial filling of the questionnaire, followed by baseline recordings, after which scent exposures alternated with the questionnaires and resting periods (Figure 1C). At the end, the final baseline was recorded. EEG and ECG data were recorded during the scent exposures and resting, but not during the questionnaire taking.

### 2.4 Electrophysiological data analysis

Data preprocessing and analysis were conducted using Python libraries: MNE 1.3.1, SciPy 1.10.0, BioSPPy 2.1.2, and pyHRV 0.4.1.

To remove power line noise, a 50-Hz notch filter was applied to the EEG and ECG data. The EEG signals were band-pass filtered in the 1–40 Hz range using a fourth order Butterworth filter. The ECG signals were high-pass filtered with a 0.1 Hz cutoff using a fourth order Butterworth filter.

For EEG preprocessing, noisy channels were noticed by visual inspection and replaced by an interpolation from the nearby channels using spherical spline interpolation. Ocular artifacts were removed with independent component analysis (ICA), using Fp1-Fp2 channels as electrooculography (EOG) references.

Spectral analysis was performed using Welch’s method with a Hamming window. The EEG power spectra were computed for the 4–25 Hz range using a 2-s interval for windowing and 8-s intervals for FFT computing. Powers within theta (4–7 Hz), alpha (8–13 Hz), and beta (15–25 Hz) frequency ranges were calculated for each lead as ratios to the power within the 4–25 Hz span, providing data for comparison across conditions.

The ECG R-peaks were identified using the BioSPPy Python library (function biosppy.signals.ecg.ecg) and visually verified (Bota et al., 2024). Heart rate and RR-intervals were computed, with RR-intervals resampled at 4 Hz for spectral analysis using a 75-s length of each Welch segment and a 1,024-s FFT length (function pyhrv.frequency_domain.welch_psd). Low frequency (LF, 0.04–0.15 Hz), high frequency (HF, 0.15–0.4 Hz), and total power (0–0.4 Hz) bands were calculated. LF and HF bands were normalized by dividing the power in each band by the sum of powers in the LF and HF bands.

Respiratory cycles were detected (function biosppy.signals.resp.resp) and visually verified. Respiratory rates were compared across the conditions.

### 2.5 Statistical analysis

The EEG ratio indices were statistically analyzed with a permutation cluster-level paired *t*-test (function mne.stats.permutation_cluster_1samp_test) to compare theta, alpha, and beta ratios across the scents, for a total of nine permutation tests. For groups of leads with statistically significant differences, group-average power ratios were calculated and used for subsequent paired comparisons across the conditions.

Statistical comparisons of the EEG indices, heart rates and respiratory rates across scent conditions were performed using the non-parametric paired Wilcoxon test.

Scores from the STAI were statistically analyzed using the Wilcoxon tests to compare state anxiety scores across the scents and baseline conditions. Spearman’s rank correlation coefficient was used to examine the relationship between the trait anxiety scores and physiological indices.

## 3 Results

### 3.1 EEG findings

**Figure 2:**
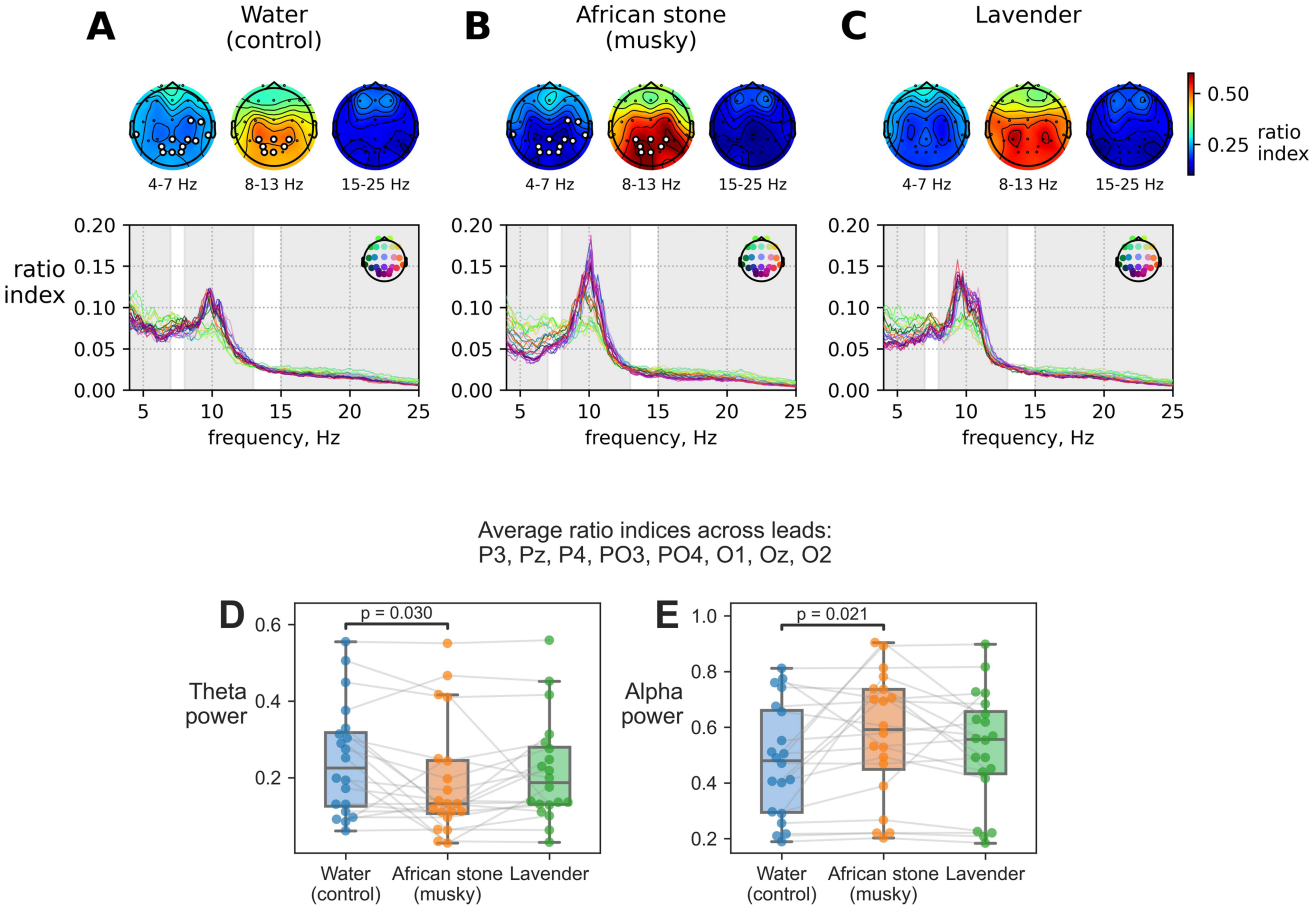
Spectra and topographic maps of EEG activity (median across participants) during scent inhalation conditions: **(A)** water, **(B)** African stone, and **(C)** lavender. White dots indicate clusters of leads showing statistically significant differences in theta and alpha ratios between African stone and water. Subplots **(D,E)** show comparisons of **(D)** theta and **(E)** alpha power across conditions, averaged over P3, Pz, P4, PO3, PO4, O1, Oz, and O2 leads. Each dot represents an individual participant; lines connect values from the same participant across conditions.

The statistical analysis of EEG spectra identified the groups of parietal-occipital leads having significant differences in theta and alpha power ratios for the comparison of inhaling water (Figure 2A) versus African stone (Figure 2B). The theta ratio index was significantly different for the comparison African stone to water (permutation cluster-level paired t-test, *p*-value = 0.036), with significant differences observed for the leads C4, T4, T6, TP9, TP10, Pz, P4, PO3, PO4, O1, Oz, and O2. The alpha ratio index was significantly different for the same comparison (*p*-value = 0.043), with differences observed for the parietal-occipital leads (P3, Pz, P4, PO3, PO4, O1, Oz). No statistically significant differences were found across conditions for the beta frequency range. No statistically significant differences were found in EEG spectra during inhalation of lavender as compared to other scents (Figure 2C).

In the subsequent analyses, theta and alpha ratios were averaged for the parietal-occipital leads (P3, Pz, P4, PO3, PO4, O1, Oz, O2) for the comparisons across different scent conditions. The non-parametric paired Wilcoxon test confirmed the decrease in theta power during African stone inhalation (Figure 2D, *W* = 47, *p*-value = 0.03) and the increase in alpha power (Figure 2E, *W* = 44, *p*-value = 0.021).

### 3.2 HRV findings

No statistically significant differences were found in the heart rate and respiratory rate across the different scent conditions.

**Figure 3:**
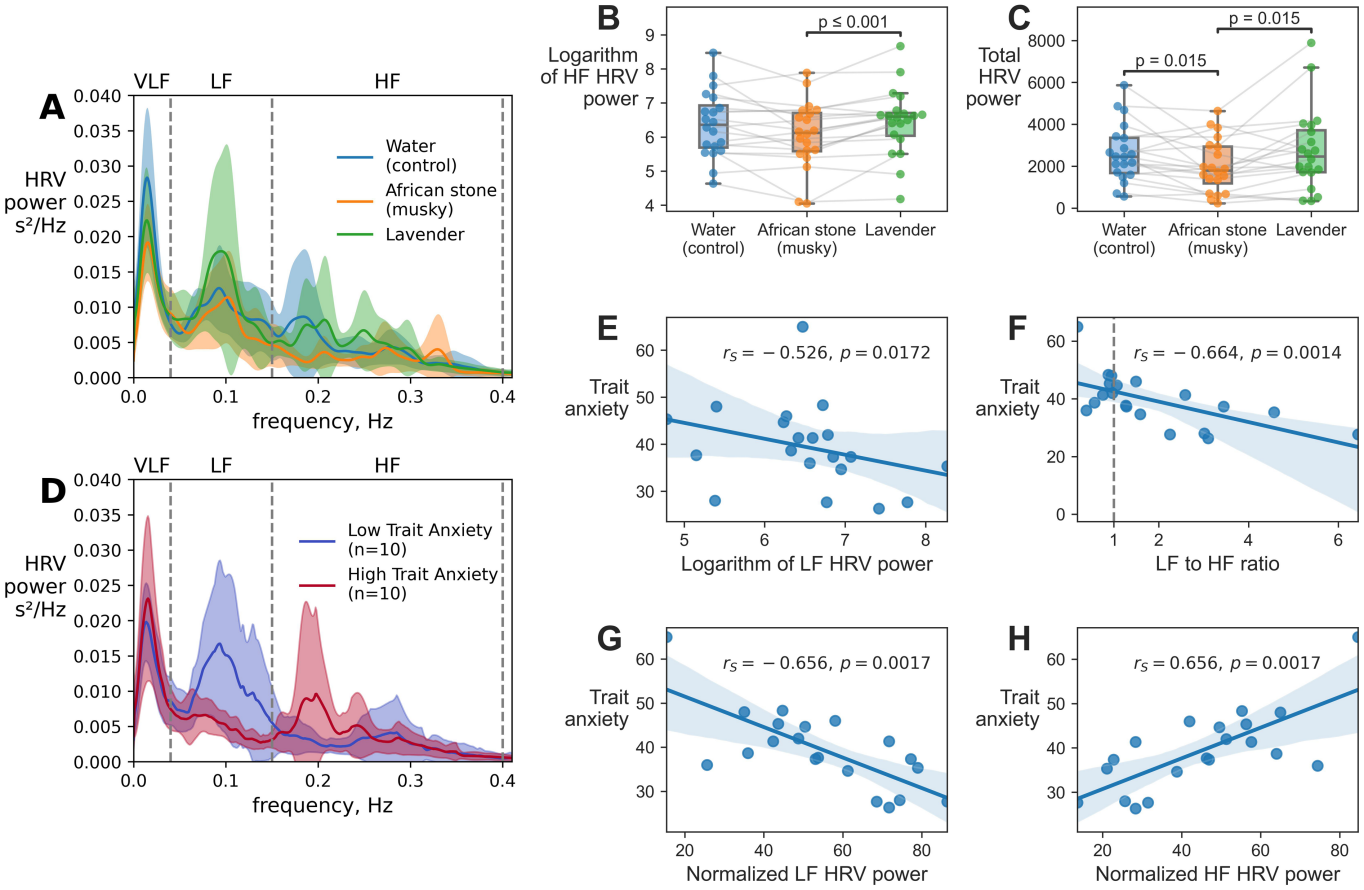
Changes in heart rate variability (HRV) across scent inhalation conditions: water, African stone, and lavender. **(A)** Average HRV spectra across conditions (mean). **(B)** High frequency HRV (HF HRV) and **(C)** total HRV power across conditions. Each dot represents an individual participant; lines connect data points from the same participant. **(D)** Average HRV spectra across participants with low and high trait anxiety scores from the STAI questionnaire (mean). Subplots **(E–H)** show relationships between trait anxiety scores and HRV indices: **(E)** logarithm of low frequency (LF) HRV power, **(F)** LF to HF ratio, **(G)** normalized LF HRV power, and **(H)** normalized HF HRV power. Each dot represents an individual participant.

Log-transformed HF HRV power and total HRV power had significant reductions during African stone inhalation as compared to lavender (Figure 3A).

Log-transformed HF HRV power was significantly decreased African during stone inhalation as compared to lavender (Figure 3B, *W* = 21, *p*-value = 0.0008). Notably, log-transformed LF HRV power had tendency toward decrease during African stone inhalation as compared to lavender that did not reach statistical significance (*W* = 53, *p*-value = 0.053).

We also found a significant decrease in total HRV power during African stone inhalation as compared to lavender (Figure 3C, *W* = 41, *p*-value = 0.015) and water (Figure 3C, *W* = 41, *p*-value = 0.015).

### 3.3 Statistical analysis of anxiety assessments

**Figure 4:**
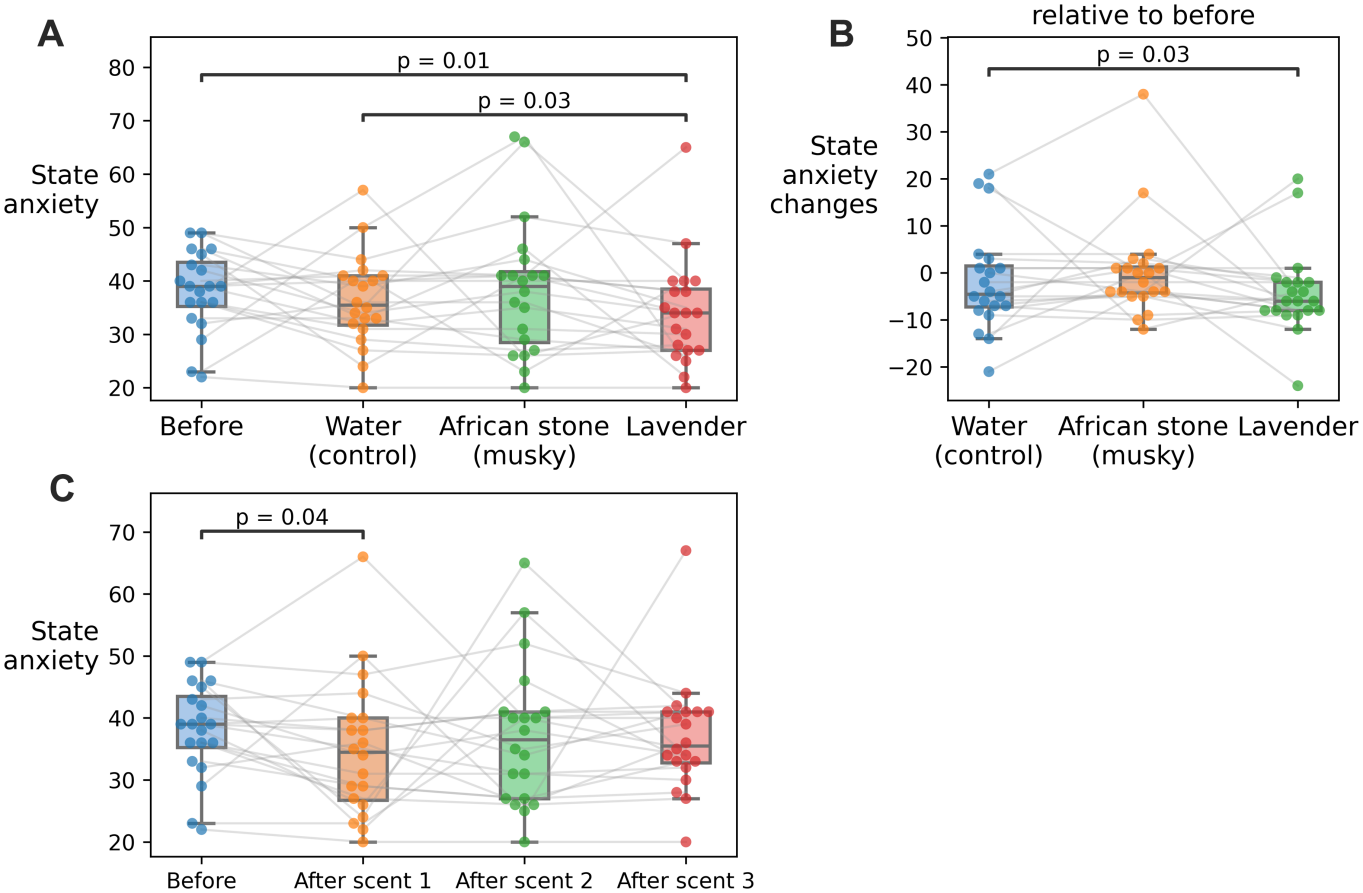
State anxiety scores from the State–Trait Anxiety Inventory (STAI) questionnaire. **(A)** State anxiety scores recorded before the experiment and immediately after each odor inhalation (four assessments in total). **(B)** Baseline-corrected changes in state anxiety following inhalation. **(C)** State anxiety scores across the experimental session timeline.

A significant reduction in state anxiety scores from the State–Trait Anxiety Inventory (STAI) was observed during lavender inhalation as compared to the baseline state anxiety scores. Lavender inhalation significantly reduced the anxiety score (Figure 4A) compared to both pre-experiment (*W* = 38.5, *p*-value = 0.012) and water conditions (*W* = 42.5, *p*-value = 0.031). Following correction of anxiety scores against baseline (pre-experiment) scores, the reduction in state anxiety after lavender exposure remained statistically significant (Figure 4B, *W* = 42.5, *p*-value = 0.031).

Notably, a general reduction in state anxiety scores was recorded over the course of the experimental session for all participants, with significant decreases after the first scent exposure compared to baseline (Figure 4C, *W* = 50.5, *p*-value = 0.043).

### 3.4 Correlational analysis

Trait anxiety scores, EEG and HRV indices during each of the scent inhalations were averaged across the assessments to investigate the correlations between trait anxiety and physiological indices.

A moderate negative correlation was found between LF HRV power and trait anxiety scores from the STAI (Figure 3D). Specifically, this was demonstrated by a negative correlation between the logarithm of LF HRV power and trait anxiety scores (Spearman’s rank correlation coefficient, *r_S_* = −0.526, *p*-value = 0.0172, Figure 3E), normalized LF HRV power and trait anxiety scores (*r_S_* = −0.656, *p*-value = 0.0017, Figure 3G), as well as between LF to HF ratio and trait anxiety scores (*r_S_* = −0.664, *p*-value = 0.0014, Figure 3F). Additionally, there was a positive correlation between normalized HF HRV power and trait anxiety scores (*r_S_* = 0.656, *p*-value = 0.0017, Figure 3H), as normalized HF power is inversely related to normalized LF HRV power. The correlation between logarithm of HF HRV power and trait anxiety scores was not statistically significant (*r_S_* = 0.23, *p*-value = 0.31).

No correlations were found between EEG indices and trait anxiety scores.

Notably, we have also found strong correlation between state and trait anxiety scores averaged for each participant across the four assessments (*r_S_* = 0.486, *p*-value = 0.03).

Table 1 shows the key results of the study, highlighting significant changes and pairwise differences with effect sizes where applicable.

**Table 1:** Summary of the study findings.

Index	Conditions, mean ± standard deviation			
Pairwise comparisons	Before	Water	African stone	Lavender
EEG: parieto-occipital ratio index				
Theta	–	**0.25 ± 0.15** ^*^ **, d = 0.36**	**0.19 ± 0.15** ^*^ **, d = 0.36**	0.22 ± 0.14
Alpha	–	**0.48 ± 0.2** ^*^ **, d = 0.43**	**0.57 ± 0.22** ^*^ **, d = 0.43**	0.53 ± 0.2
Beta	–	0.16 ± 0.09	0.14 ± 0.09	0.15 ± 0.08
ECG: low frequency (LF), high frequency (HF), total heart rate variability (HRV)				
Logarithm of LF HRV power	–	6.71 ± 0.68	6.27 ± 1.06	6.55 ± 1.09
Logarithm of HF HRV power	–	6.38 ± 0.97	**6.07 ± 0.98** ^*^ **, d = 0.26**	**6.45 ± 0.98** ^*^ **, d = 0.26**
Total HRV power	–	2634.31 ± 1390.82^*^, d^*^ **= 0.47**	2013.35 ± 1273.96^*,**^, d^*^ = 0.47, d^**^ **= 0.45**	2768.81 ± 1953.93^**^, d^**^ **= 0.45**
LF to HF ratio	–	1.85 ± 1.38	1.94 ± 1.73	1.87 ± 2.11
State–Trait Anxiety Inventory (STAI) questionnaire				
State anxiety	38.10 ± 7.62^*^, d^*^ **= 0.45**	36.4 ± 8.64^**^, d^**^ **= 0.25**	38.5 ± 12.68	34.05 ± 10.04^*,**^, d^*^ = 0.45, d^**^ **= 0.25**
Trait anxiety	40.05 ± 8.85	39.8 ± 8.77	39.85 ± 9.28	38.65 ± 10.24
Correlational analysis				
Normalized LF HRV power vs. trait anxiety	**r**_**S**_ **= −0.656, *p* = 0.0017**			

Mean ± standard deviation values for EEG indices, ECG-based HRV measures, and State and Trait Anxiety Inventory (STAI) scores. Statistically significant changes are shown in bold. Asterisks indicate statistically significant differences between specific conditions in pairwise comparisons. The absolute value of Cohen’s *d* is also reported for comparisons that reached statistical significance.

## 4 Discussion

This study demonstrated that both lavender and African stone influenced the subjective and physiological measures of anxiety, but in different ways (Table 1). In line with the previous reports, lavender inhalation led to a significant reduction in self-reported anxiety scores (Donelli et al., 2019; Karan, 2019). By contrast, African stone mostly affected the physiological measures, according to both EEG and ECG findings. Specifically, African stone exposure reduced theta power and increased alpha power in parieto-occipital regions, along with the decreases in HF and total HRV powers.

Reduction in the total HRV is conventionally interpreted as a sign of increased arousal or stress, regardless of the valence of the experienced emotional state. Likewise, a decrease in HF HRV is typically interpreted as reduced parasympathetic modulation, which is consistent with the interpretation that African stone produced an arousing physiological effect. Thus, the best explanation for the observed decreases in HF and total HRV during African stone inhalation appears to be a heightened arousal and its corresponding autonomic effects.

The EEG changes observed with African stone are more nuanced to interpret. While increased parieto-occipital alpha activity could be interpreted as relaxation, calling this a straightforward indicator of reduced anxiety is problematic given the concurrent HRV results. A different explanation could be given to this result: the alpha rhythm increased because attention shifted toward olfactory processing and away from visual and somatosensory processing (Van Diepen et al., 2019). Indeed, the musky and unfamiliar scent of African stone may have elicited a particular engagement compared to the more familiar scent of lavender.

Regarding theta activity, its functional significance depends strongly on cortical origin. Frontal midline theta activity could reflect anxiety-related vigilance and cognitive control (Cavanagh and Shackman, 2014), whereas parieto-occipital theta activity would indicate drowsiness and low arousal. In this study, the reduction in parieto-occipital theta is consistent with the shift toward a more alert and wakeful state rather than increased anxiety. This interpretation is supported by the concurrent increase in alpha activity and reductions in HF and total HRV. Overall, these physiological responses are best interpreted as enhanced attention to the unfamiliar scent accompanied by autonomic arousal.

Lavender inhalation in our study significantly reduced self-reported anxiety scores, which agrees with the previous reports (Donelli et al., 2019; Karan, 2019). However, the absence of accompanying physiological effects suggests that reduced self-reported anxiety is mostly subjective. Most studies on lavender’s anxiolytic properties have focused primarily on self-reported anxiety, which consistently showed a reduction, as highlighted in the systematic review and meta-analysis by Donelli et al. (2019). In contrast, relatively few studies examined physiological outcomes, and those that did predominantly relied on the ECG-derived measures. Reductions in blood pressure (Bahrami et al., 2017; Bikmoradi et al., 2015; Hashemi and Faghih, 2018) and heart rate (Bahrami et al., 2017; Grunebaum et al., 2011; Matsumoto et al., 2017) were reported, and some studies documented increases in HF HRV resulting from lavender aromatherapy (Igarashi, 2013; Matsumoto et al., 2013, 2017). Notably, two studies did not find any significant changes in the ECG-derived indices (Burnett et al., 2004; Franco et al., 2016). Findings from the EEG studies are even more heterogeneous, including a report of increased beta power interpreted as heightened alertness (Diego et al., 1998) and a report of increased theta and alpha power interpreted as relaxation (Sayorwan et al., 2012). Most studies where physiological effects of lavender were reported had inhalation intervals lasting longer than 10 min and were often repeated multiple times. We had shorter, 5-min intervals of lavender exposure, so it is possible that this duration was insufficient to elicit measurable physiological responses. Overall, the scarcity and inconsistency of EEG findings in aromatherapy research further suggest more studies should be undertaken to clarify issues like the proper odor-exposure time. At this point we acknowledge the possibility that relatively brief exposure to lavender smell is sufficient to produce a subjective feeling of relaxation, but the neural effects are subtle and difficult to discern from the other EEG and HRV effects related to the ongoing changes in alertness and attention.

Even though subjective and physiological measures of anxiety level are not necessarily equally expressed, as we see from the present findings, physiological still informative work. Thus, negative correlation was prominent between LF HRV and trait anxiety. This relationship aligns with the previous findings summarized in the systematic review by Cheng et al. (2022). Even though lavender inhalation increased HRV only slightly and this effect was statistically insignificant (Figure 3A), this tendency occurred for the same HRV spectral band where participants with lower trait anxiety had increased HRV power (Figure 3D), which suggests that lavender’s subtle physiological effects are of the same nature as the ones found in the individuals with lower anxiety.

The divergence observed between the subjective and physiological markers of anxiety highlights the value of a multimodal approach in assessing aromatherapy outcomes. Relying solely on self-reports may miss important physiological changes. At the same time, conventional EEG and HRV indices can be affected by a wide range of emotional and cognitive factors, such as general arousal, attentional engagement, or fatigue. Together, these considerations point to a broader challenge in anxiety research: the need to refine, and perhaps reconsider, traditional biomarkers to more accurately reflect the specificity of acute anxiety responses, especially in the context of sensory interventions.

Based on our current findings, we propose that different scents could modulate anxiety through distinct mechanisms. Lavender appears to strongly reduce perceived anxiety, while African stone appears to heighten physiological arousal. Adding more aromas to this equation could enable the creation of tailored, multifaceted aromatherapy protocols designed to target physiological arousal and/or subjective distress, depending on individual needs and therapeutic goals.

## 5 Conclusion

This pilot study demonstrated that lavender and African stone modulated anxiety-related measures in different ways. Lavender inhalation produced a clear reduction in self-reported anxiety, consistent with prior literature, but did not elicit measurable changes in EEG or ECG indices, possibly due to the short (5 min) exposure duration. In contrast, African stone primarily influenced physiological measures, reducing parieto-occipital theta power, increasing alpha power, and decreasing HF and total HRV power, which is consistent with increased arousal. The changes in EEG during exposure to African stone likely reflect increased attention to an unfamiliar scent, rather than merely relaxation.

The divergence between subjective and physiological responses highlights the need for multimodal assessment in aromatherapy studies. Self-reports may overlook meaningful physiological changes, while conventional EEG and HRV indices can be influenced by the processes unrelated to anxiety. The proposed multimodal approach will improve tailoring aromatherapy interventions to properly target perceived anxiety and/or physiological changes.

## 6 Limitations

While this study provides valuable insights into the differential effects of lavender and African stone scents on subjective and physiological markers of anxiety, several limitations warrant consideration.

First, the sample size of 20 participants, while common in psychophysiological studies, limits the generalizability of these findings. Future research should aim to replicate these results in larger and more diverse populations to confirm the consistency of the effects observed here.

Second, the study’s reliance on EEG and HRV as primary physiological markers of anxiety introduces challenges in interpreting these data as definitive indicators of short-term state anxiety. EEG indices, such as theta and alpha power, and HRV measures, including HF and total HRV powers, reflect broader arousal and autonomic responses that may not be exclusively linked to anxiety. Further research with additional physiological markers (e.g., skin conductance, cortisol levels) could provide a more comprehensive understanding of the short-term effects of olfactory stimuli on anxiety.

Additionally, the divergence observed between subjective and physiological measures of anxiety highlights a potential limitation in using self-reported assessments to capture rapid or subtle shifts in anxiety state. The State–Trait Anxiety Inventory (STAI) and similar scales may not fully reflect the immediate impact of sensory interventions, such as scent exposure, especially in high-stress environments like dental offices. Future studies might benefit from using real-time, moment-to-moment anxiety tracking tools or exploring alternative self-assessment methods to capture transient effects more accurately.

Moreover, it is important to consider that participants’ responses to lavender could have been influenced by cultural and individual expectations, as lavender is widely known and advertised as a calming scent. Such prior beliefs or associations could contribute to a placebo-like effect on self-reported anxiety, potentially amplifying the subjective calming response independently of any direct physiological impact. This factor highlights the challenge of disentangling true scent-induced effects from learned or suggestive influences, underscoring the need for future studies to include control conditions that better account for expectancy effects and participants’ prior experiences with specific aromas.

Finally, individual differences in olfactory sensitivity, scent preferences, and baseline anxiety levels could have influenced participants’ responses to the scents. While measures were taken to control for some of these factors, these individual differences likely contributed variability to the results. Future research should address this by stratifying participants based on their olfactory sensitivity and scent preference profiles or by using standardized scent exposure levels.

By acknowledging these limitations, we hope to encourage further exploration of scent-based interventions in clinical settings, with an emphasis on refining both methodology and measures to enhance understanding and applicability in anxiety management.

## Data Availability

The raw data supporting the conclusions of this article will be made available by the authors, without undue reservation.
